# Age-Specificity of Clinical Dengue during Primary and Secondary Infections

**DOI:** 10.1371/journal.pntd.0001180

**Published:** 2011-06-21

**Authors:** Khoa T. D. Thai, Hiroshi Nishiura, Phuong Lan Hoang, Nga Thanh Thi Tran, Giao Trong Phan, Hung Quoc Le, Binh Quang Tran, Nam Van Nguyen, Peter J. de Vries

**Affiliations:** 1 Division of Infectious Diseases, Tropical Medicine and AIDS, Academic Medical Center, Amsterdam, The Netherlands; 2 Center for Infection and Inflammation Research (CINIMA), Academic Medical Center, University of Amsterdam, Amsterdam, The Netherlands; 3 Theoretical Epidemiology, University of Utrecht, Utrecht, The Netherlands; 4 PRESTO, Japan Science and Technology Agency, Saitama, Japan; 5 Tropical Diseases Clinical Research Center, Cho Ray Hospital, Ho Chi Minh City, Vietnam; 6 Department of Virology, Cho Ray Hospital, Ho Chi Minh City, Vietnam; 7 Binh Thuan Medical College, Phan Thiet City, Vietnam; Universidade de São Paulo, Brazil

## Abstract

**Background:**

This study aims to estimate the age-specific risks of clinical dengue attack (i.e., the risk of symptomatic dengue among the total number of dengue virus (DENV) infections) during primary and secondary infections.

**Methods:**

We analyzed two pieces of epidemiological information in Binh Thuan province, southern Vietnam, i.e., age-specific seroprevalence and a community-wide longitudinal study of clinical dengue attack. The latter data set stratified febrile patients with DENV infection by age as well as infection parity. A simple modeling approach was employed to estimate the age-specific risks of clinical dengue attack during primary and secondary infections.

**Results:**

Using the seroprevalence data, the force of infection was estimated to be 11.7% (95% confidence intervals (CI): 10.8–12.7) per year. Median age (and the 25–75 percentiles) of dengue fever patients during primary and secondary infections were 12 (9–20) and 20 (14–31) years, respectively. The estimated age-specific risk of clinical dengue increases as a function of age for both primary and secondary infections; the estimated proportion of symptomatic patients among the total number of infected individuals was estimated to be <7% for those aged <10 years for both primary and secondary infections, but increased as patients become older, reaching to 8–11% by the age of 20 years.

**Conclusions/Significance:**

For both primary and secondary infections, higher age at DENV infection was shown to result in higher risk of clinical attack. Age as an important modulator of clinical dengue explains recent increase in dengue notifications in ageing countries in Southeast Asia, and moreover, poses a paradoxical problem of an increase in adult patients resulting from a decline in the force of infection, which may be caused by various factors including time-dependent variations in epidemiological, ecological and demographic dynamics.

## Author Summary

Although age at dengue virus (DENV) infection is recognized as playing a key role in characterizing the risks of clinical attack and disease severity, the contributions of age to disease development have yet to be quantified in detail. We estimated the age-specific risk of clinical attack (i.e., the risk of symptomatic dengue among the total number of DENV infections) during primary and secondary DENV infections in Vietnam, by employing a simple epidemiological modeling approach in which two pieces of epidemiological data sets were used, i.e., (i) age-specific seroprevalence and (ii) age-specific frequency of clinical attack of dengue during primary and secondary infections. We showed that those at higher age are more likely to develop symptomatic dengue than younger individuals for both primary and secondary infections; the estimated proportion of symptomatic patients among the total number of infected individuals was below 7% for those aged younger than 10 years of age for both primary and secondary infections, but was shown to be elevated as the patients become older, reaching to 8–11% by the age of 20 years. Age as an important modulator of clinical dengue attack explains recent increase in dengue notifications in ageing countries in Southeast Asia, and moreover, poses a paradoxical problem of an increase in adult patients resulting from a decline in the force of infection, which may be caused by various factors including time-dependent variations in epidemiological, ecological and demographic dynamics.

## Introduction

Dengue ranks among the most important infectious diseases with a major impact on public health in many countries in the tropics and subtropics. Estimates showed that approximately 3.5 billion people, ∼55% of the world's population live in countries at risk for dengue [1]. The global incidence has increased steadily over the last six decades, simultaneously with an increase in geographic distribution and a transition from epidemic-type dengue with long interepidemic intervals to endemic-type with seasonal fluctuation [2]–[4].

Dengue virus (DENV) transmission primarily takes place through bites by the principal mosquito vectors, *Aedes aegypti*, which feed preferentially on human blood, and are often found in and around human dwellings [5], [6]. Infection with any of the four dengue serotypes results in either asymptomatic infection, or a spectrum of clinically apparent disease ranging from mild undifferentiated febrile illness to severe dengue of which dengue shock syndrome (DSS) is the most common life threatening syndrome in children [7]. The mechanisms for the variable clinical outcome are not completely elucidated, but genetic factors, race, maternal antibody, circulating serotype and infection with multiple serotypes are believed to play an important role in determining the disease severity [8].

When it comes to the disease severity, a well-established epidemiological risk factor is the age at infection [9]–[12]. It is known that differences in clinically apparent dengue vary by age; pre-school children and infants have rather more often undifferentiated febrile illnesses while pre-adolescent children often develop fever [13], and moreover younger children with dengue hemorrhagic fever (DHF) are known to experience more severe clinical outcome (e.g. higher case fatality ratio) than adults [12]. Dengue is a pediatric disease in Southeast Asia except for Singapore. A rigorous vector control program has substantially reduced the transmission in Singapore and, as a consequence, dengue patients are predominantly seen in adults. Nevertheless, with the elevation in patients' age, the outcome of DENV infection may be more favorable since most adult dengue patients present with dengue fever (DF) instead of with DHF [14].

Apart from age, infection parity is known to be a critical factor of disease severity; primary infection with any of the four serotypes is believed to elicit lifelong immunity against that serotype, but confers partial or transient immunity against other serotypes [15], [16]. Cross-reactive, but sub-neutralizing DENV-reactive IgG, acquired by a previous heterotypic serotype infection may enhance DENV infectivity which may result in higher viral burden and contribute to induced disease severity. Heterologous secondary infections have been associated with large, clinical outbreaks of DHF/DSS where severe dengue occurs most frequently in children [12]. In some rigorous observations, the age group with highest susceptibility to contracting DSS was that of children with a modal age of 8 to 11 years [17], [18].

Although age at infection and infection parity are the representative key modulators of clinical dengue and disease severity, their relationship has yet to be established and explicitly quantified. A previous study investigated the relationship between age at primary infection in Brazil and the risk of febrile illness, suggesting that adults are more likely than children to have clinical dengue [19]. This result should ideally be validated in the Southeast Asian settings. Moreover, we have yet to understand the age-specific risk of symptomatic disease during secondary infection. This present study tackles these issues by analyzing epidemiological data sets in southern Vietnam, focusing specifically on the age-specific risk of symptomatic dengue given infection. That is, we do not consider age-specific severity of clinical dengue, and rather, focusing only on the conditional probability of illness given infection. Because of high transmission potential with co-circulating multiple serotypes, dengue has been mainly a pediatric disease in Vietnam, and ironically, this provides us with an opportunity to investigate the age-specific risks of clinical attack both during primary and secondary infections. The present study aims to characterize a fundamental relationship between age at DENV infection and the risk of developing clinical attack.

## Methods

### Ethics statement

The protocols for recruitment, testing and follow-up were approved by the Review Board of the Cho Ray Hospital, Ho Chi Minh City, Provincial Health Services and the community stations. The study was explained and discussed in meetings (e.g. with the People's Committee of the communities, the PHC-staff and school teachers). All patients (or, for children, the parents or guardian) gave written informed consent.

### Study site

Our study rests on empirical observations in Binh Thuan province which is located along the south-eastern coast of Vietnam, 150 km northeast of Ho Chi Minh City, wedged between the Truong Son forested mountains (alt. 1100–1642 m) in the west and the South Chinese Sea in the east. It covers 7,828 km^2^ and the estimated population was 1,140,429 inhabitants in 2004. The majority of the population lives in rural areas, with approximately 187,042 people in and around the capital, Phan Thiet City. Healthcare is provided by a provincial hospital in Phan Thiet city, nine district hospitals and 115 community posts for primary healthcare (PHC) and disease control.

### Epidemiological data

We examined two pieces of epidemiological information, (i) age-specific seroprevalence and (ii) age-specific frequency of clinical attack of dengue during primary and secondary infections, as determined by serological confirmation, in order to estimate the age-specific risk of clinical dengue attack. Figure S1 shows the participating PHCs and the villages in which the serosurvey were conducted. The mean distance between the source of seroprevalence data and all PHCs was 40.5 km (range 3–87 km).

The former data set included age stratified seroprevalence data from a cross-sectional survey among primary school children in two communities (Ham Kiem and Ham Hiep). This survey was conducted among 961 children, aged from 7 to 14 years, in 2003. The detailed results are given elsewhere [20], [21]. Approximately 1 ml of blood was collected by finger puncture in plain vials (Greiner, Minicollect), left to clot at ambient temperature, centrifuged at 1000 rpm for 15 minutes and serum was transferred to a sterile vial for storage at −20°C until testing. All samples were tested for the presence of dengue specific serum antibodies against dengue virus using a commercial available indirect IgG enzyme-linked immunosorbent assay (ELISA). The indirect IgG ELISA was performed according to the manufactures instructions (Focus Technologies Inc., Cypress, CA, USA) [22]. Optical density (OD) values were measured at 450 nm with 620 nm as a reference with a Benchmark microplate reader (Bio-Rad Laboratories, Inc., Hercules, CA, USA). Results were expressed as the ratio between the sample OD value and the OD value of the kit calibration serum (ODR), both after subtraction of the OD of an enclosed blank specimen. ODR values >1 were considered positive. Seroprevalence (i.e. proportion positive) was stratified by age group.

Second, age-specific frequencies of clinical attack were derived from a prospective longitudinal observational study at 12 PHCs across Binh Thuan province and at the provincial malaria control center in Phan Thiet City. That study examined the etiology of acute undifferentiated fever (AUF) from March 2001 to March 2006 [23]–[27]. All patients presenting with AUF were included. AUF was defined as any febrile illness of duration less than 14 days, confirmed by an axillary temperature of ≥38.0°C, without any clinical indication for either severe systemic or organ specific disease. Malaria was excluded by microscopic examination of a thick blood smear. A standardized questionnaire was employed to collect demographic and clinical information. Serum samples were collected from patients with a febrile illness by venous puncture on presentation (acute sample; t0) and after 3 weeks (convalescence sample; t3). Serum samples were stored at −20°C at the study sites until monthly transfer to Cho Ray hospital (Ho Chi Minh City, Vietnam), where they were stored at −70°C. Complete sets of acute and convalescence samples were selected for dengue serology. In 2001 all collected serum pairs were tested with dengue ELISA; afterwards paired samples were randomly selected as two patients per PHC and per month from the total data set [23]. Paired serum samples were tested for dengue with direct IgG ELISA and IgM-Capture ELISA (Focus Technologies Inc., Cypress, CA, USA). It should be noted that these serological tests did not distinguish between an infection with DENV from that with Japanese Encephalitis virus (JEV). Cross-reactivity with JEV antibodies may have occurred, potentially involving a small proportion of samples [21]. Details regarding the ELISA and the interpretation of results were described previously [28]. In brief, a fourfold increase of antibody concentrations between t0 and t3 was considered significant. The IgM concentration on t3, relative to the IgG concentration on t3 was also used as a criterion. Acute primary DENV infection was defined as positive IgM on t3 with an IgM/IgG ratio on t3 greater than one. A positive IgM on t3 with an IgM/IgG ratio on t3 less than one, or a negative IgM reaction on t3 but with a positive IgG t3 and a fourfold molar increase of IgG between t0 to t3 were classified as acute secondary dengue. A negative IgM reaction on t3, a positive IgG on t3 but without a fourfold increase between t0 and t3 was classified as “not acute dengue but past infection”, and a subject of both negative IgM and IgG on t3 was classified as “no dengue”. It could well be possible that patients with an immune response to secondary dengue infection have had a tertiary or even dengue infection with a fourth serotype. Because the immune response between sequential dengue infections was not explicitly distinguished by using this ELISA, we grouped all individuals with serological indicative of a repeated infection and defined these as the secondary dengue infections in the present study. The epidemiological, virological and clinical features have been described elsewhere [29]. All four serotypes have been circulating during the study period with a potential shift of the dominant DENV serotype over time. Whereas DENV-4 was the dominant serotype in 2001–2002, DENV-1 and DENV-2 later came to be most frequently isolated.

### Mathematical model and statistical estimation

#### Sequential transmission dynamics of DENV infection

**Figure 1:**
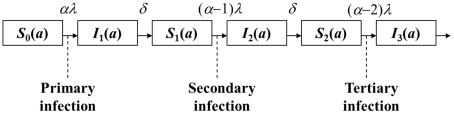
Sequential transmission dynamics of DENV infection. Each compartment represents an age-specific state of DENV infection. *S*_i_(*a*) is the proportion of susceptible individuals who are at risk of infection by the serotypes that remain (*α*-*i*) after previous infections at age *a* (for *i* = 0, 1 and 2). *I*_i_(*a*) represents the proportion of those who have experienced infections with *i*-th serotype and remain still protected against the remaining heterologous serotypes due to short-lived cross-protective immunity. At age 0, *S*_0_(0) = 1 and other compartments are 0. *λ* is the force of infection, i.e. the rate at which susceptible individuals experience infection, for a single serotype. *α* is a scaling factor which can be interpreted as the number of co-circulating serotypes. We assume that human hosts experience an identical risk of infection between heterologous serotypes. *δ* is the rate at which cross-protective immunity against remaining heterologous serotypes declines. Infection with a fourth serotype is ignored, because it is very uncommon.

A simple epidemiological model was developed to estimate the age-specific risks of clinical dengue attack during primary and secondary infections. The model was constructed to describe the age-specific frequencies of primary and secondary infections (which were used as the denominator population, representing infected individuals), and we combine it with our empirical data of the age-specific frequencies of individuals with symptomatic dengue during primary and secondary infections (which were dealt with as the numerator population, representing symptomatic dengue patients) in order to estimate the age-specific conditional risk of clinical dengue attack (given infection). Figure 1 illustrates the compartments of our model, describing the lifetime risks of primary, secondary and tertiary infections. The model accounts for the age-specificity of infection and acquisition of serotype-specific immunity over lifetime. Similar sequential assumptions have been employed in previous studies [30]–[33]. The mathematical descriptions are given in Text S1. Among the parameters in Figure 1, we estimate the force of infection (i.e., the rate at which susceptible individuals get infected), *λ*, from seroprevalence data, and the remaining parameters, i.e., the scaling factor representing the number of co-circulating serotypes (*α*) and the loss rate of cross-protective immunity (*δ*), were assumed known. All four serotypes have been observed in Binh Thuan province, but the relative frequency of serotype 3 has been smaller than other serotypes [29], [34]. Because of the irregularity in the serotype-specific frequency, which yields variations in the transmission potential between different serotypes, we therefore varied *α* from 2.5 to 4.0 with a default value 3.5. The duration of cross-protective immunity in literature ranges from 1–2 weeks [35] to 2–9 months [36]. Although not based on an explicit statistical estimation, the latter study suggested that within 2 months after a primary attack offered full protection, and that within 9 months after the primary attack may still yield partial protective immunity to infection with heterologous strain (e.g. partial reductions in clinical symptoms) [36]. Accordingly, we varied *δ* from 10 days to 1 year with a default value of 1 month. Due to absence of strain information, we ignored potential variations in virulence between strains (see Discussion). The following assumptions were made to attain a simple statistical estimation; (i) the force of infection, *λ*, was age-independent (and we focused on relatively young age groups in the population), (ii) the transmission intensities has reached to an endemic equilibrium so that the time-inhomogeneity can be ignored (as we focused on the data sets covering a short period of time), (iii) the transmission potential is identical among co-circulating serotypes (though we accounted for non-integer values for *α* as a theoretical adjustment), (iv) once an individual acquires infection with a single serotype, he/she becomes temporarily immune against infection with other heterologous serotypes (which is lost at a rate *δ*) and permanently immune against further infections with the identical serotype and (v) our empirical data represent the transmission dynamics for the entire population of Binh Thuan province. Although omitted from Figure 1 for simplicity, the natural death rate, *μ* (per year), occurs in each compartment. We obtained the population data from the Binh Thuan statistics office, Phan Thiet City, southern Vietnam, and estimated *μ*, assuming that the population is stable and also that the survivorship in this rural population is sufficiently approximated by an exponential distribution.

The age-specific proportion of those having experienced infection at least with one serotype is given by 1−exp(−*αλa*), and the age-specific survivorship at age *a* is written as exp(−*μa*). Supposing that there were *n*_a_ seropositive and *m*_a_ seronegative results at age *a*, the likelihood function to estimate *λ* is
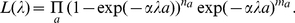
(1)Exactly the same argument was made to estimate *μ*.

#### Estimation of the age-specific risk of clinical attack

Subsequently, we estimated the age-specific risks of clinical attack during primary and secondary infections, *r*_1_(*a*) and *r*_2_(*a*), respectively. Let *S*_0_(*a*), *S*_1_(*a*) and *S*_2_(*a*) be the expected proportions of susceptible individuals (among a total population), based on our model, who are susceptible to the remaining *α*, *α*−1 and *α*−2 serotypes at age *a*, respectively. The incidence of primary, secondary and tertiary infections at age *a* are then given by *αλS*_0_(*a*), (*α*−1)*λS*_1_(*a*) and (*α*−2)*λS*_2_(*a*), respectively. We ignored infection with fourth serotype assuming that it is rare, and we grouped the incidence of secondary and tertiary infections to adhere to our empirical observation of secondary infections in the longitudinal survey (and assumed that the risk of clinical attack between secondary and tertiary infections is identical). Accordingly, *r*_1_(*a*) and *r*_2_(*a*) were, respectively, conditioned on primary infection and secondary and tertiary infections. Observing a signature of an increase in *r*_1_(*a*) and *r*_2_(*a*) as a function of age in the empirical data, three statistical models, logit (*l*), Weibull (*w*) and exponential (*e*) distributions, were employed for these functions, i.e.,
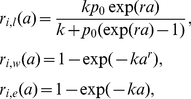
(2)for *i* = 1 or 2, where *k*, *p*_0_ and *r* are the parameters. Given a data set of *n* patients during primary infection and *m* patients during secondary infection with their age at infection *a*_i_, the likelihood function to estimate parameters for *r*_1_(*a*) and *r*_2_(*a*) is
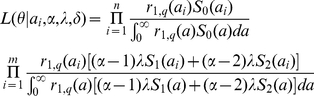
(3)for *q* = *l*, *w* or *e*. It should be noted that *S*_i_(*a*) were solved analytically and replaced by parameters *μ*, *λ*, *α* and *δ* (see Text S1). Maximum likelihood estimates of the parameters were obtained by minimizing negative logarithm of (3). The lack-of-fit of three parametric models were compared by employing Akaike information criterion (AIC).

## Results

### Demography and the force of infection

**Figure 2:**
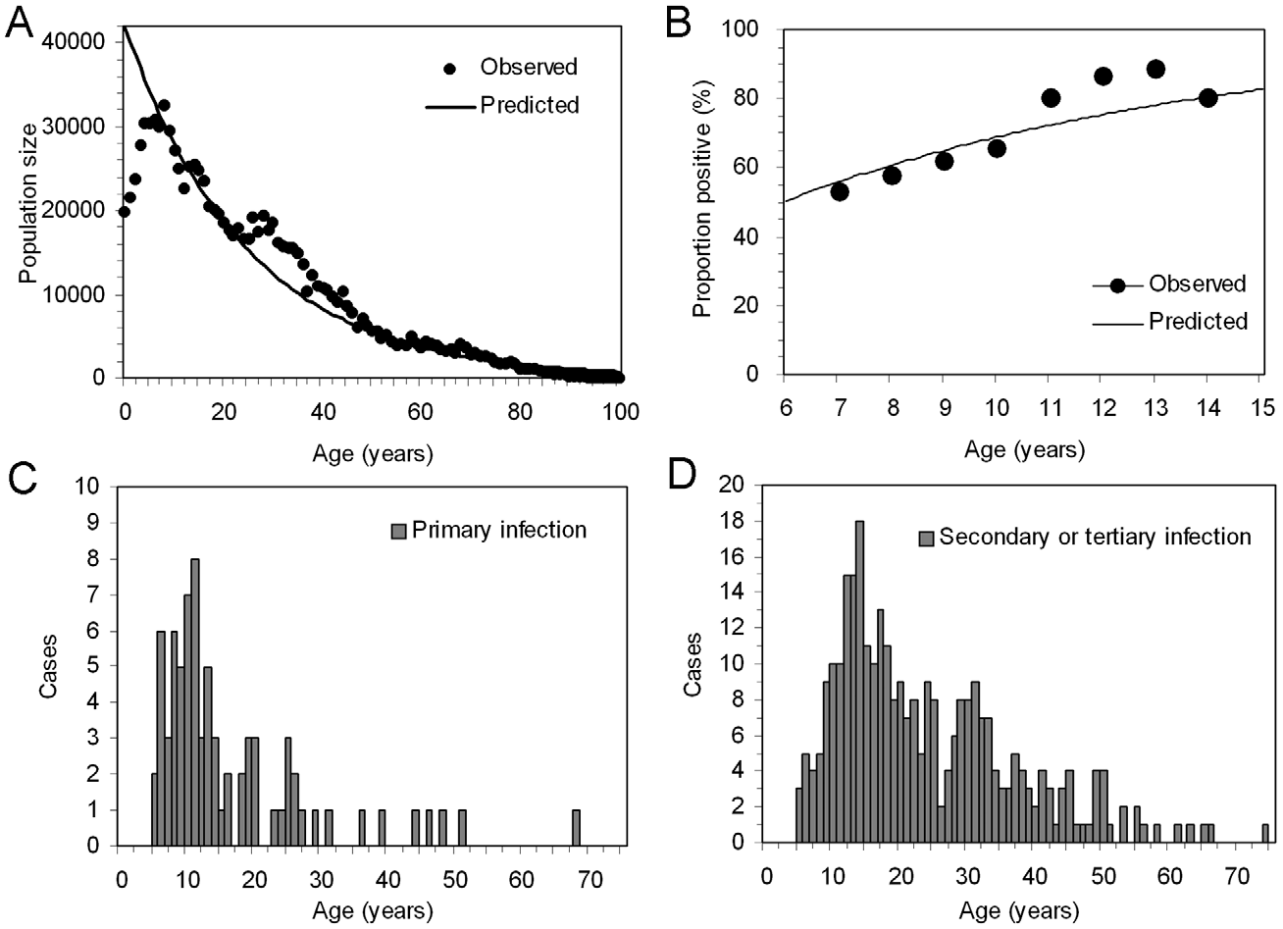
Population and the epidemiological data of DENV infection in Binh Thuan province, southern Vietnam. (A) Age-specific population size (circles). The continuous line shows the predicted age-distribution by employing an exponential distribution as the survivorship function. (B) Age-specific seroprevalence against at least one serotype of dengue among those aged from 7–14 years (data source; [20]). The continuous line shows the predicted proportion positive using the maximum likelihood estimate of the force of infection (i.e. 11.7% per year). (C& D) Observed absolute numbers of clinical attack of dengue during (C) primary and (D) secondary or tertiary infections. We assume that D does not include any symptomatic dengue subjects due to infection with fourth serotype.

The age-specific population size of Binh Thuan province is shown in Figure 2A. Mean and median ages (and the 25–75 percentiles) were 24.7 and 20 (9–35) years, respectively. Employing an exponential approximation, the natural death rate (*μ*) was estimated to be 4.05×10^−2^ (95% confidence interval (CI): 4.04×10^−2^–4.05×10^−2^) per year. Figure 2B shows the observed and predicted age-dependent seroprevalence. The force of infection of the total of *α* serotypes, *αλ*, was estimated as 11.7% (95% CI: 10.8–12.7) per year.

### Symptomatic primary, secondary or tertiary dengue infections

A total of 14595 febrile patients were included in our longitudinal survey. Eighty-three patients were excluded as the inclusion criteria for AUF were not met. That is, eleven patients were afebrile (i.e. <38°C), axillary temperatures of 19 patients were not documented, and 53 patients were diagnosed with an organ specific disease (e.g. pharyngitis) at presentation. Paired sera were collected from 8268 febrile patients; 1938 (23.4%) serum pairs were tested with dengue IgM- and IgG-ELISA. Of these, DENV infection was serologically confirmed in 382 patients (19.7%). Primary infection accounted for 76 confirmed patients (19.9%), and secondary infection for 306 patients. Their age-specific frequencies are shown in Figures 2C and 2D, respectively. Median age (and the 25–75 percentiles) of dengue fever patients during primary and secondary infections were 12 (9–20) and 20 (14–31) years, respectively, revealing that secondary infection occurs at significantly older ages (p<0.01, Wilcoxon test). None of confirmed dengue infections were suggestive of severe clinical forms of dengue, DHF or DSS.

### Epidemiological dynamics of dengue

**Figure 3:**
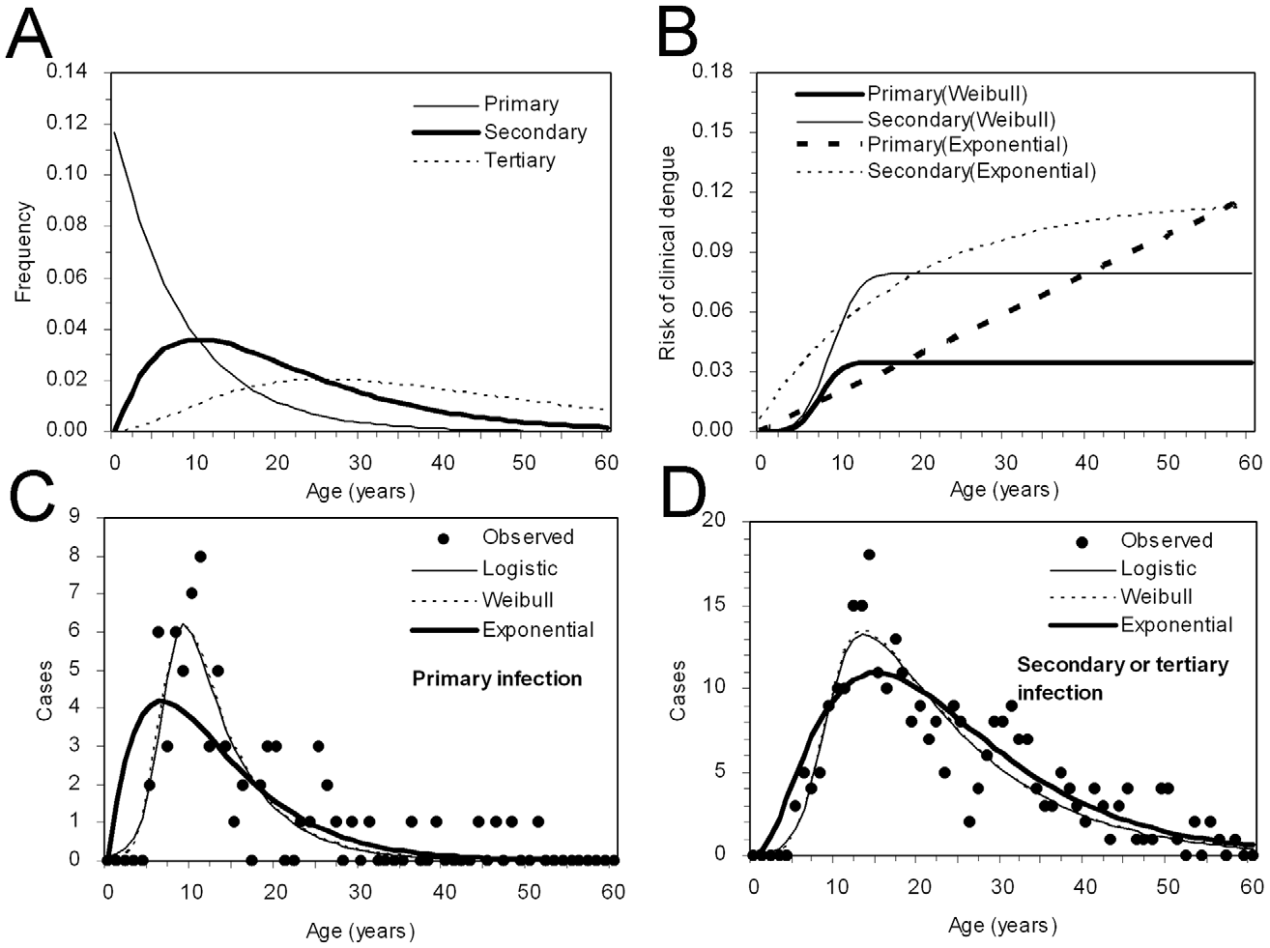
Epidemiological dynamics of dengue infection. (A) Age-specific frequency of primary (thin solid line), secondary (thick solid line) and tertiary infections (dashed line). The frequencies are characterized by the force of infection, the mean duration of cross-protective immunity and the number of co-circulating serotypes. (B) Age-specific probability of developing clinical attacks of dengue given primary (thick lines) and secondary or tertiary infection (thin lines). The continuous lines represent the results employing Weibull model, while broken lines are the estimates based on exponential model. (C & D) Comparisons between observed and expected age-specific numbers of clinical attacks during (C) primary and (D) secondary or tertiary infections. Three different models, logit (thin continuous lines; *k* = 0.200, *p*_0_ = 0.224×10^−3^, *r* = 0.971), Weibull (dashed lines; *k* = 0.259×10^−3^, *r* = 3.991) and exponential (thick continuous lines; *k* = 0.324×10^−4^) approaches were employed.

Using the maximum likelihood estimate of *αλ* = 0.117 and the default values of *α* and *δ*, age-specific frequencies of primary, secondary and tertiary infections are shown in Figure 3A. As indicated by 1/*αλ*, the mean age at primary infection is 8.5 years, and secondary and tertiary infections occur at older ages. Figure 3B shows the estimated risks of symptomatic dengue during primary and secondary infections for Weibull and exponential assumptions (results with logit model is not shown as it yielded the similar qualitative pattern to Weibull). The conditional risks of clinical attack were shown to increase as a function of age during both primary and secondary infections. The estimated proportion of symptomatic subjects among the total number of infected individuals was below 7% for those aged younger than 10 years of age for both primary and secondary infections, but increased as patients become older, reaching to 8–11% by the age of 20 years. Within the age-band examined (<60 years), both assumptions indicate that the risk of symptomatic dengue during secondary infection is higher than that during primary infection for all ages. The Weibull distributed age-specific risk of clinical dengue during primary and secondary infections plateau around the ages of 15 and 19 years, respectively. Figures 3C and 3D compares the observed and predicted age-specific numbers of symptomatic subjects during primary and secondary infections, respectively. Using default values of *α* and *δ*, the AIC values were estimated to be 1461, 1460 and 1470 for logit, Weibull and exponential assumptions, respectively. The preference of Weibull assumption did not change when we varied *α* from 2.5 to 4.0 and *δ* from 10 days to 12 months.

**Figure 4:**
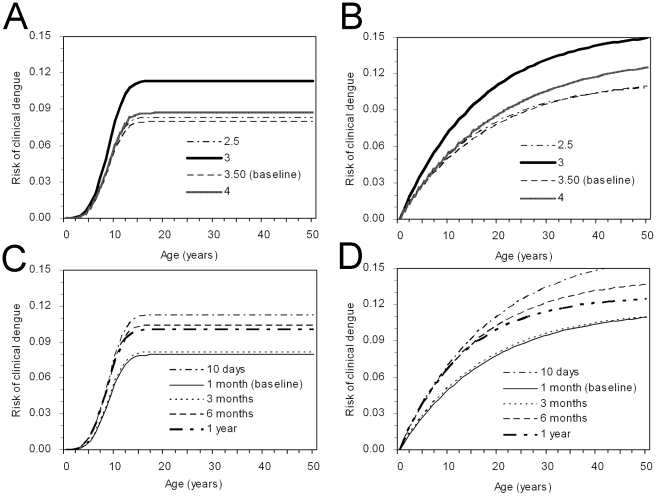
Sensitivity of the conditional risk of symptomatic dengue disease given infection to uncertain parameters. All panels examine the age-specific risks of developing clinical attacks of dengue given secondary or tertiary infection. (A & C) Weibull model. (B & D) Exponential model. A & B examines the sensitivity of the risk to different number of co-circulating serotypes (*α*, the range: 2.5 and 4.0). We assume that human hosts experience an identical risk of infection between serotypes. The baseline value of *α* was set at 3.5. C & D examines the sensitivity of the probability to different mean durations of cross-protective immunity following primary and secondary infections (1/*δ*, the range: 10 days to 1 year). The duration of cross-protection following primary infection was assumed as identical to that following secondary infection. The baseline value of 1/*δ* was set at 1 month. Results from the logit model are not shown, because the qualitative patterns are similar to those of Weibull model. Also, the sensitivity of the risk of symptomatic disease during primary infection is not shown, because the probability is not sensitive to variations in *α* and *δ*.

Figures 4A and 4B examine the univariate sensitivity of *r*_2_(*a*) to *α* for Weibull and exponential assumptions, respectively. The age-specific risk of clinical attack was the highest for all ages with *α* = 3, but the overall difference in the conditional risk from those with other *α* remained within 5% for Weibull assumption. The Weibull assumption was always preferred in terms of AIC, but the difference in AIC between the logit and Weibull models remained <3 for the range of *α* that we examined. Similarly, Figures 4C and 4D show the univariate sensitivity of *r*_2_(*a*) to *δ* for the Weibull and exponential assumptions, respectively. The age-specific risk of symptomatic secondary dengue infection with default *δ* (1 month) yielded the smallest estimates, but again the difference in the estimated risks of clinical attack remained within 5% for Weibull assumption. Again, AIC values indicated Weibull model as the best, but the differences in AIC values between logit and Weibull models remained <3 for the whole range of *δ*. Since our prospective study involved only 29 and 36 children aged 10 years or younger during primary and secondary infections, respectively, we also examined the effect of sample size on the age-specific conditional risk of illness given infection. Even when the absolute numbers of children ≤10 years was doubled, the qualitative patterns of risks (i.e. age-specific increase in the risk of disease, and higher risk during secondary infection than primary infection) remained the same. However, the age at which the risk of symptomatic dengue is saturated with logit and Weibull assumptions, was shifted to the left, approximately by 3–4 years younger as compared to the baseline. The AIC values were estimated to be 1664, 1664 and 1686 for logit, Weibull and exponential assumptions, respectively. These increases perhaps reflect a mismatch of the estimated force of infection with the incidence data.

## Discussion

We estimated the age-specific risks of clinical dengue attack by combining two epidemiological data sets, (i) age-specific seroprevalence and (ii) age-specific frequency of symptomatic dengue during primary and secondary infections. The former data set was used to reconstruct the age-specific frequencies of primary and secondary infections (including those with and without symptoms), and subsequently, by taking the age-specific ratio of the latter data set to the reconstructed infection frequency with an aid of modeling method, the age-specific conditional probability of disease given infection was estimated. Although our model required a number of simplifying assumptions, we have shown unambiguously that the conditional risks of clinical attack increased as a function of age for both primary and secondary infections. Thus, higher age-groups, e.g., adolescents and young adults are more likely to develop symptomatic dengue than younger individuals, e.g., primary school children. Moreover, Weibull model indicates that the age-specific risks of symptomatic disease in adults both during primary and secondary infection remain almost independent of age, perhaps reflecting greater variations in age-specific susceptibility to symptomatic disease among children and adolescents. To our knowledge, the present study is the first to characterize the age-specific risk of developing clinical attack during both primary and secondary dengue infection by means of epidemiological modeling method. Whereas the risk of severe complications given clinically apparent dengue (e.g., the risk of hospitalization and the case fatality ratio) in children is higher than in adults [12], the age-specific risk of disease itself is the other way around and increases with age.

Although pathogenesis of DENV infection and its severe complications involves many unanswered questions, and despite their multifactoral nature, our study emphasizes a critical importance of age as a key modulating factor of the risks of clinical attack during primary and secondary infections. Two important practical implications can be drawn from our results. First, as was shown in a study in Thailand, a rapid demographic transition has taken place in many Southeast Asian countries where swift ageing, i.e., the shift of the age distribution of human population toward older ages, has been observed [37]. Our results support the notion of Cummings et al. [37] in that the ageing society is truly at risk of increase in dengue incidence. Indeed, Binh Thuan province yielded an average age at infection of 8.5 years, which is slightly greater than a previous published estimate (e.g. 5.2–6.1 years in Rayong, Thailand, 1980 [31]), indicating that the transmission is less intensive in Binh Thuan province in the 21st century than in Thailand, 1980. Second, while the incidence in Southeast Asian countries has been increasing [38]–[42], there has been a decline in the force of infection, resulting in a shift in the age distribution of DHF toward older age groups [33], [37]. In addition to ageing, the decline might have reflected various factors including time-dependent variations in epidemiological, ecological and demographic dynamics, e.g natural decline in the transmission, successful control of vectors and human migration. Our exercise suggests that such a decline in the force of infection could nevertheless result in an increase in older symptomatic individuals, thereby resulting in a paradoxical increase in the total number of symptomatic dengue patients (and thus, the incidence of symptomatic dengue individuals for the entire population). Although clinical outcome of severe DENV infection in adults may be more favorable than in children, in terms of prognosis of clinically apparent dengue [12], [14], the incidence of symptomatic cases may not decrease with a slight decline in the force of infection. Clarification on the population impact of age-specific risks of clinical attack on the total number of severe forms of dengue is the subject of our future studies.

Despite our successful estimation of the age-specific risk of clinical dengue attack, two limitations of the present study should be noted. First, the majority of DENV infections remain asymptomatic, and a very small amount of symptomatic patients (∼5%) results in severe disease [9]. Our longitudinal survey data did not capture sub-clinical infections or very mild symptomatic patients, implying that our estimate of the risk of clinical attack may have been potentially underestimated. Nevertheless, our survey did not select for specific signs and symptoms of dengue, examining only AUF patients by laboratory testing, and thus, we believe that the age distributions (Figures 2C and 2D) reflected unbiased age-specific frequencies of all the symptomatic subjects during primary and secondary infections. Second, we assumed that the force of infection is time- and age-independent and the transmission potential is identical among all co-circulating serotypes. Ignorance on these realisms, e.g., seasonality, age-dependency and decline in *λ* over a long period of time, forces us to accentuate the lack of precision in our estimates of *r*_1_(*a*) and *r*_2_(*a*). For example, the lack of age-dependency may have led to slight underestimation in *r*_1_(*a*) among small children and potentially an overestimation for both *r*_1_(*a*) and *r*_2_(*a*) among adults. Besides, whereas modeling approach such as ours certainly requires a number of unrealistic assumptions, we believe that our conclusion on the qualitative pattern, i.e., an increase in the risk of clinical attack with age, remains intact.

Of course, various other pre-infection factors other than age and infection parity contribute to the risk of disease severity, including cross-protective immunity between serotypes, the number of co-circulating serotypes and their pathogenicity [40],[43]–[49]. Indeed, our incidence data did not include any DHF patients among a total of 306 symptomatic patients during secondary infection. Although no direct comparison can be made, this 0% is significantly smaller than that estimated in a prospective study in Thailand [9]. Possible explanations are that (a) our prospective study focused on the etiology of AUF, and the fraction of patients with severe dengue manifestations (e.g. who may not have presented at the PHCs or have sought care directly at higher medical level) might have been potentially disregarded, (b) the average age at infection in Binh Thuan province is higher than that in Bangkok during 1980s, and secondary infection at higher age can reduce the absolute number of DHF patients, and (c) not only serotype but also different strains could yield differential virulence given symptomatic infection. Moreover, molecular epidemiological studies have demonstrated that long-term expansion of dengue epidemic is regulated by selection-driven adaptive evolution of DENV strains [50]–[52]. Since the absolute risk of symptomatic infection is vulnerable to the virus (strain)-specific virulence as well as our reliance on symptoms of patients and help seeking behavior, the estimate of absolute risk could potentially vary from one region to another. In that sense, our simple approach is regarded as a first step to characterize the epidemiological determinants of dengue in a rudimentary fashion, and our study at least confirmed age-specific increase in the risk of clinical dengue given infection, offering practically important implications. Modeling with sequential infection assumption still remains to be a common strategy to capture the serotypic sequential infection mechanisms [31]–[33], [49], and future incorporation of strain-specificity needs to account for strain specific virulence as well as host-response (including cross immunity) to each strain [53], which will be far more complex than the simplistic sequential approach.

In conclusion, we examined the age-specific risks of clinical attack during primary and secondary DENV infections in Vietnam, showing that those at higher age-group are more likely to develop symptomatic disease than younger individuals. Age as an important modulator of clinical dengue attack explains recent epidemiological shift in dengue notification in ageing countries in Southeast Asia, and moreover, poses a paradoxical problem of an increase in adult patients resulting from a decline in the force of infection which may be caused by various factors including time-dependent variations in epidemiological, ecological and demographic dynamics.

## Supporting Information

Figure S1 **Map of Vietnam (left) and Binh Thuan province (right).** Participating PHCs in communes are indicated with numbers and sources of seroprevalence data are indicated with letters. 1: Me Pu; 2: Huy Khiem; 3: Tra Tan; 4: Tan Minh; 5: Tan Xuan; 6: Ham My; 7: Duc Long; 8: Ham Tien; 9: Ham Phu; 10: Phan Tien; 11: Binh Tan; 12: Vinh Hao. A: Ham Kiem; B: Ham Hiep.
(TIF)

Text S1 **Mathematical description of the epidemiological model.**
(DOC)

Alternative Language Abstract S1 **Translation of the abstract into Vietnamese by Hoang Lan Phuong.**
(DOC)

Checklist S1 **STROBE checklist.**
(DOC)
