# Supplementary figures and images for: Age-Specificity of Clinical Dengue during Primary and Secondary Infections
Source: PLoS Negl Trop Dis. 2011 Jun 21;5(6):e1180. doi: 10.1371/journal.pntd.0001180 (PMC3119638; doi:10.1371/journal.pntd.0001180)

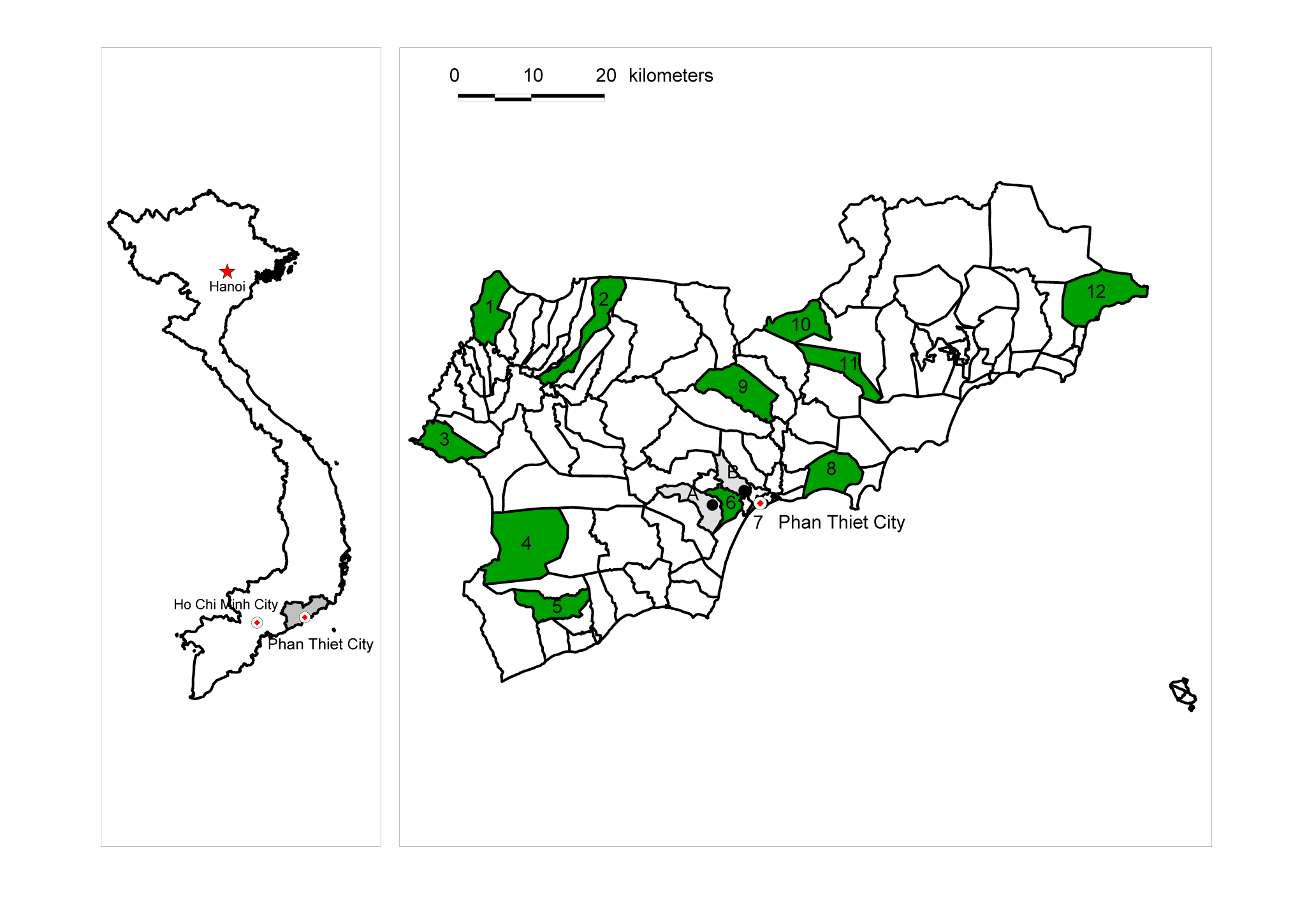

Supplement: Figure S1 — Map of Vietnam (left) and Binh Thuan province (right). Participating PHCs in communes are indicated with numbers and sources of seroprevalence data are indicated with letters. 1: Me Pu; 2: Huy Khiem; 3: Tra Tan; 4: Tan Minh; 5: Tan Xuan; 6: Ham My; 7: Duc Long; 8: Ham Tien; 9: Ham Phu; 10: Phan Tien; 11: Binh Tan; 12: Vinh Hao. A: Ham Kiem; B: Ham Hiep. (TIF) [file pntd.0001180.s001.tif]
