# Supplementary material for: Age-Specificity of Clinical Dengue during Primary and Secondary Infections
Source: PLoS Negl Trop Dis. 2011 Jun 21;5(6):e1180. doi: 10.1371/journal.pntd.0001180 (PMC3119638; doi:10.1371/journal.pntd.0001180)
Supplement: Text S1 — Mathematical description of the epidemiological model. (DOC) [file pntd.0001180.s002.doc]

**Mathematical description of the epidemiological model**

Here we describe the details of model that we employed for statistical inference (Figure 1). Among the total population, the proportion of individuals who are susceptible to (*α*-*i*) serotypes at age *a* are denoted by *S*_i_(*a*) (for *i* = 0, 1 and 2 with a default value *α* = 3.5). Similarly, let *I*_i_(*a*) represent the proportion of those who have experienced infections with *i*-th serotype and remain still protected against the remaining heterologous serotypes at age *a* due to short-lived cross-protective immunity. Supposing that the force of infection is age- and time-independent *λ*, the sequential transmission dynamics as a function of age *a* are described by the following differential equations:

$$\begin{matrix} \frac{dS_{0}(a)}{da}=-(\alpha\lambda+\mu)S_{0}(a), \\ \frac{dI_{1}(a)}{da}=\alpha\lambda S_{0}(a)-(\delta+\mu)I_{1}(a), \\ \frac{dS_{1}(a)}{da}=\delta I_{1}(a)-((\alpha-1)\lambda+\mu)S_{1}(a), \\ \frac{dI_{2}(a)}{da}=(\alpha-1)\lambda S_{1}(a)-(\delta+\mu)I_{2}(a), \\ \frac{dS_{2}(a)}{da}=\delta I_{2}(a)-((\alpha-2)\lambda+\mu)S_{2}(a), \\ \frac{dI_{3}(a)}{da}=(\alpha-2)\lambda S_{2}(a)-\mu I_{2}(a). \end{matrix}$$

where *α* is a scaling factor of co-circulating serotypes (interpreted as the number of co-circulating serotypes) and *δ* is the rate at which *I*_i_(*a*) looses the cross-protective immunity against remaining heterologous serotypes. We estimate *λ* from seroprevalence data, and assume that *α* and *δ* are known.

Our longitudinal survey included neither mild symptomatic dengue in infants nor vascular permeability syndrome among infants due to maternal antibody, and we ignore maternal antibody during first half year of life for simplicity. We assume that *S*_0_(0) = 1 and other compartments are 0 at age 0. Accordingly, the age-specific incidence of primary, secondary and tertiary infections at age *a* are given by *αλS*_0_(*a*), (*α*-1)*λS*_1_(*a*) and (*α*-2)*λS*_2_(*a*), respectively, and we analytically solved the equation system (4) to replace *S*_i_(*a*) by parameters *μ*, *λ*, *α* and *δ*. Supposing that the age-specific conditional probabilities of clinical dengue attack given primary and secondary infections are *r*_1_(*a*) and *r*_2_(*a*), respectively, the age-specific frequencies of primary infection and a combination of secondary and tertiary infections with clinical attack are expressed as *r*_1_(*a*)*αλS*_0_(*a*) and *r*_2_(*a*)[(*α*-1)*λS*_1_(*a*)+(*α*-2)*λS*_2_(*a*)], respectively. To estimate the parameters for *r*_1_(*a*) and *r*_2_(*a*), we normalized these frequencies, i.e.,

$$f_{1}(a)=\frac{\alpha\lambda r_{1}(a)S_{0}(a)}{\int_{0}^{\infty} \alpha\lambda r_{1}(s)S_{0}(s)ds}=\frac{r_{1}(a)S_{0}(a)}{\int_{0}^{\infty} r_{1}(s)S_{0}(s)ds}$$

and

$$f_{2or3}(a)=\frac{r_{2}(a)[(\alpha-1)\lambda S_{1}(a)+(\alpha-2)\lambda S_{2}(a)]}{\int_{0}^{\infty} r_{2}(s)[(\alpha-1)\lambda S_{1}(s)+(\alpha-2)\lambda S_{2}(s)]ds}.$$

We used (5) and (6) for the likelihood equation (3) in the main text.
