# Supplementary material for: Age-Specificity of Clinical Dengue during Primary and Secondary Infections
Source: PLoS Negl Trop Dis. 2011 Jun 21;5(6):e1180. doi: 10.1371/journal.pntd.0001180 (PMC3119638; doi:10.1371/journal.pntd.0001180)
Supplement: Alternative Language Abstract S1 — Translation of the abstract into Vietnamese by Hoang Lan Phuong. (DOC) [file pntd.0001180.s003.doc]

**Đặc trưng theo tuổi trong nhiễm dengue tiên phát và thứ phát trên lâm sàng**

*Tóm tắt*

*Nền tảng: Nghiên cứu này nhằm đánh giá những nguy cơ đặc trưng theo tuổi của bệnh nhân bị sốt dengue trên lâm sàng (nghĩa là nguy cơ của bệnh về triệu chứng trong tổng số bệnh nhiễm virus dengue) trong nhiễm tiên phát và thứ phát trên lâm sàng.*

*Phương pháp: Chúng tôi phân tích 2 mảng thông tin dịch tễ học ở tỉnh Bình Thuận, miền nam Việt nam, gồm tần suất nhiễm virus dengue dựa vào huyết thanh học đặc trưng theo tuổi và một nghiên cứu cắt dọc trên cộng đồng bệnh nhân bị sốt dengue trên lâm sàng. Dữ liệu sau này phân tầng bệnh nhân sốt do nhiễm virus dengue theo tuổi cũng như nhiễm bệnh trước đó . Một tiếp cận theo mô hình đơn giản được chọn để đánh giá những nguy cơ đặc trưng theo tuổi của sốt dengue trên lâm sàng trong nhiễm tiên phát và thứ phát.*

*Kết quả: Sử dụng dữ liệu tần suất nhiễm virus dengue dựa vào huyết thanh học, tỷ lệ mắc bệnh (force of infection) được đánh giá là 11.7% một năm (95% độ tin cậy: 10.8-12.7). Tuổi trung bình (và 25-75 phần trăm trong nhóm) của bệnh nhân nhiễm dengue tiên phát là 12 (9-20) và thứ phát là 20 (14-31). Nguy cơ đặc trưng theo tuổi của sốt dengue trên lâm sàng được đánh giá gia tăng như một chức năng của tuổi trong cả sốt tiên phát và thứ phát. Tỷ lệ đánh giá được của những ca bệnh có triệu chứng trong tổng số những cá nhân bị nhiễm bệnh được đánh giá là < 7% ở trẻ < 10 tuổi cả trong sốt dengue tiên phát và thứ phát nhưng tăng lên ở bệnh nhân lớn hơn, lên tới 8-11 % ở bệnh nhân 20 tuổi.*

*Kết luận / Ý nghĩa: Trong sốt dengue tiên phát và thứ phát, tuổi nhiễm virus dengue càng cao dẫn đến nguy cơ cao của các diễn tiến bệnh trên lâm sàng. Tuổi là một điều biến quan trọng của sốt dengue trên lâm sàng, điều này giải thích cho tình trạng gia tăng những ca bệnh được báo cáo gần đây trong các quốc gia có tuổi thọ cao ở Đông Nam Á, và hơn nữa, đặt ra một vấn đề nghịch lý của sự gia tăng số bệnh nhân người lớn từ sự tỷ lệ mắc bệnh (force of infection) mà có thể gây ra do những yếu tố khác nhau bao gồm những biến thiên phụ thuộc thời gian trong động lực dịch tễ học, sinh thái học và nhân khẩu học.*
